# Epidemiology of strongyle nematode infections and first report of benzimidazole resistance in *Haemonchus contortus* in goats in South Darfur State, Sudan

**DOI:** 10.1186/s12917-019-1937-2

**Published:** 2019-06-04

**Authors:** Khalid M. Mohammedsalih, Amna Khalafalla, Ahmed Bashar, Adam Abakar, Abdelhakaim Hessain, Fathel-Rahman Juma, Gerald Coles, Jürgen Krücken, Georg von Samson-Himmelstjerna

**Affiliations:** 1 Faculty of Veterinary Science, University of Nyala, P.O. Box 155, Nyala, Sudan; 2 Faculty of Veterinary Medicine, University of Khartoum, P.O. Box 32, Khartoum North, Sudan; 3 Faculty of Medical Laboratory Sciences, University of Gezira, P.O. Box 20, Wadmedani, Sudan; 4 Ubley Biologics, P.O. Box 170, Bristol, Ubley BS40 6JA UK; 5 Institute for Parasitology and Tropical Veterinary Medicine, Freie Universität Berlin, Robert-von-Ostertag-Str. 7-13, 14163 Berlin, Germany

**Keywords:** Albendazole, Resistance, *Haemonchus contortus*, Goats, South Darfur state, Sudan

## Abstract

**Background:**

Since pastoralists in South Darfur, Sudan, had complained about lack of albendazole (ABZ) efficacy to control nematodes in goats, the frequency of infection with gastrointestinal helminths was studied before in vivo faecal egg count reduction tests (FECRT) were conducted using ABZ orally either at the dose recommended for sheep, 5 mg/kg body weight (bw) or at 10 mg/kg bw. Experiments included goats naturally infected with gastrointestinal nematodes or experimentally infected with local *Haemonchus contortus* isolates. Three study areas (Nyala, Beleil and Kass) were visited in autumn or winter.

**Results:**

Out of 478 screened goats, 82.4% were infected with gastrointestinal helminths and 82% were shedding eggs of strongyle nematodes with 90% of the strongyle larvae representing *Haemonchus* spp. A FECRT using naturally infected goats (*n* = 225: 71 untreated, 104 and 50 treated with 5 and 10 mg ABZ/kg bw, respectively) detected reduced ABZ efficacy in Nyala and Kass. Paired and unpaired FECRT calculations detected reductions of 72–92% with samples taken at 8 days post treatment with 5 mg ABZ/kg bw and of 85–94% with 10 mg ABZ/kg bw. The FECRT based on day 14 post treatment samples showed reductions of 69–77% with 5 mg/kg and of 75–87% with 10 mg ABZ/kg bw. In Beleil, ABZ efficacy was 95%. In the egg hatch test EC_50_ values for Nyala and Kass ranged from 0.12–0.24 μg thiabendazole/ml, corresponding to benzimidazole resistant phenotypes. Only *Haemonchus* spp. larvae were present after treatments in coprocultures. When the efficacy was evaluated experimentally using isolates of *H. contortus* from Nyala and Kass, the 5 mg ABZ/kg dose revealed reductions of 76–78% on day 8 and of 62–70% on day 14 with the unpaired method. Using 10 mg ABZ/kg, the FECR was still only 77–82%.

**Conclusions:**

Both, in vivo and in vitro methods detected resistant *H. contortus* populations in goats from South Darfur State. The time point 14 days post treatment was more sensitive for detection of ABZ resistance than 8 days post treatment. This is the first report on the occurrence of anthelmintic resistance in Sudan confirming that anthelmintic resistance selection is occurring in African subsistence farming systems.

**Electronic supplementary material:**

The online version of this article (10.1186/s12917-019-1937-2) contains supplementary material, which is available to authorized users.

## Background

Goats are an important resource for poor communities, since they are able to survive in hardy conditions with high temperatures, low humidity and minimal available feed [1]. In South Darfur State, Sudan, livestock farming includes about 1.67 million goats making it one of the most important states in Sudan for animal production providing substantial income for pastoralists and the government [2, 3].

In almost all countries where sheep and goats are kept, parasitic nematodes pose a serious threat to animal health and production. The economic impact inflicted due to the infection with these parasites is high even in subsistence farming systems and has e.g. been estimated in Ethiopia to be several million dollars annually [4]. In tropical areas, such as Sudan, the most common nematode genera known to affect sheep and goats are *Haemonchus* spp., *Trichostrongylus* spp., *Cooperia* spp., *Nematodirus* spp. and *Oesophagostomum* spp., with the highest pathogenic effect caused by *Haemonchus contortus* [5–7]. Control of these parasites has been performed for several decades by the routine and often frequent use of anthelmintics, with the practical use of three major classes: benzimidazoles (e.g. albendazole (ABZ)), imidazothiazoles (e.g. levamisole) and macrocyclic lactones (e.g. ivermectin) [8]. The effective strategic control of gastrointestinal nematode (GIN) infections also requires understanding of the prevalence of these parasites within the given environment and the risk factors that are associated with their transmission [9]. The three mentioned anthelmintic classes have been commonly used in Sudan to control helminth infections of ruminants for more than two decades, and they have been distributed under various trade names by different companies without strategic plans for their use [10, 11]. In a recent study on echinococcosis, also questionnaire data on the use of anthelmintics in South Darfur State, southwest Sudan, was published [11]. All three above mentioned anthelmintic classes were found to be used over the year in the treatment of GIN infected goats. In this study, 40% of the interviewed farmers treated goats with ABZ in summer, while this percentage was reduced to 27 and 6% in autumn and winter, respectively. Treatment of goats with ivermectin was reported by 11, 31 and 33% of the farmers in winter, autumn and summer, respectively. Levamisole was found to be used only at a minor level (4%). Since these drugs have been used for a long time, their activity in the field may be reduced due to the development of resistance [12]. However, no reports of resistance development have been published for Sudan yet. Previous studies conducted in sheep and goats and in different parts of Sudan, including South Darfur, showed faecal egg count reduction (FECR) efficacies of anthelmintics (i.e. ABZ) to be in the range of 95 to 100% [2, 10, 13].

Since the metabolism of ABZ is different in sheep and goats, the latter require a higher (double) dose than sheep [14]. However, in many countries, including Sudan (Mohammedsalih, personal observation), the package labels for ABZ are identical for sheep and goats explicitly stating to use a 5 mg ABZ/kg body weight (bw) for goats [9, 15]. Therefore, the use of this drug in goats, according to the package label at a standard dosage for sheep, corresponds to a constant drug under-dosing and reduces its efficacy [14]. This may explain why nematodes resistant to ABZ were more frequently detected in goats than sheep [9, 16].

During the recent past, in South Darfur some producers have been complaining that ABZ has declined in efficacy, especially in goats (Mohammedsalih, personal observation). Therefore, the objectives of this study were to provide basic epidemiological data regarding infections with gastrointestinal helminth in goats and to investigate whether the perceived change of ABZ efficacy in goats in South Darfur State is due to treatment failure because of under-dosing or the actual development of benzimidazole resistance. In order to estimate both, the efficacy of the typically used dosage (5 mg ABZ/kg bw, sometimes repeated after 14 days) and the presence of resistant populations, effects of 5 mg ABZ/kg bw and 10 mg ABZ/kg bw were compared.

## Results

### Prevalence of gastrointestinal helminths

The frequency of infection with gastrointestinal helminths in goats in the three study areas of South Darfur (Nyala, Beleil and Kass) was 82.4% as determined using the Mini-FLOTAC protocol. The identified helminths were strongyle nematodes, *Strongyloides papillosus* and *Moniezia* spp. When faecal cultures for differentiation of strongyle L3 were examined, the results revealed mixed infections with three groups: *Haemonchus* spp., *Trichostrongylus* spp. and Chabertidae (*Oesophagostomum* spp.*/Chabertia* spp.), with *Haemonchus* spp. (90%) showing the highest percentage (Table 1). The infection rate with strongyle nematodes was 82% while only 0.4% of the tested animals were detected positive for *Moniezia* spp. In Kass, an unusually high infection rate (9.4%) with both strongyles and *S. papillosus* was observed. Moreover, an unusually high infection rate of 8.5% with both strongyles and *Moniezia* spp. was found in young goats (Table 2). The rate of simultaneous co-infection with all three helminth groups was very low (0.6%) (Tables 1 and 2).

**Table 1:** Prevalence, arithmetic mean egg counts (range) and coprocultures (%) of gastrointestinal helminths in the faeces of naturally infected goats at Nyala (Domaia and Majok), Beleil and Kass, South Darfur State, Sudan, using Mini-FLOTAC technique

	All animals	Season		Study area				
				Nyala			Beleil	Kass
				Domaia	Domaia	Majok		
		Autumn	Winter	Autumn	Winter	Winter	Autumn	Autumn
Prevalence of the infection								
No. of the tested goats	478	343	135	117	105	30	55	171
No. (%) of the infected goats	394 (82.4)	297 (86.6)	97 (71.9)	114 (97.4)	75 (71.4)	22 (73.3)	49 (89.1)	134 (78.4)
Strongyles	392 (82)	296 (86.3)	96 (71.1)	114 (97.4)	74 (70.5)	22 (73.3)	49 (89.1)	133 (77.8)
Strongyles + *Strongyloides papillosus*	20 (4.2)	19 (5.5)	1 (0.7)	0 (0)	0 (0)	1 (3.3)	3 (5.5)	16 (9.4)
Strongyles + *Moniezia* spp.	23 (4.8)	19 (5.5)	4 (3)	4 (3.4)	2 (1.9)	1 (3.3)	7 (12.7)	9 (5.3)
Infection with all three parasites	3 (0.6)	3 (0.9)	0 (0)	0 (0)	0 (0)	0 (0)	1 (1.8)	2 (1.2)
*Moniezia* spp.	2 (0.4)	1 (0.3)	1 (0.7)	0 (0)	1 (1)	0 (0)	0 (0)	1 (0.6)
Goats shedding ≥500 strongyle epg	251 (52.5)	221 (64.4)	30 (22.2)	102 (87.2)	20 (19.1)	10 (33.3)	40 (72.7)	79 (46.2)
Egg count/gram of positive faeces								
Strongyles	1842 (20–31,040)	2241 (40–31,040)	625 (20–5440)	2699 (120–22,080)	553 (20–5440)	867 (80–5200)	1543 (40–7320)	2100 (40–31,040)
*Strongyloides papillosus*	332 (10–1600)	342 (10–1600)	120	0	0	120	140 (80–320)	387 (10–1600)
Coprocultures for strongyles (%)								
*Haemonchus* spp.	90	87	95	75	99	90	96	91
*Trichostrongylus* spp.	6	10	6	24	1	10	2	4
*Oesophagostomum* spp.*/Chabertia* spp.	2	3	0	1	0	0	2	5

**Table 2:** Prevalence and arithmetic mean egg counts (range) of gastrointestinal helminths in the faeces of naturally infected goats of different sex and age groups using Mini-FLOTAC technique

	All animals	Sex		Age	
		male	female	young	adult
Prevalence of the infection					
No. of the tested goats	478	50	428	118	360
No. (%) of the infected goats	394 (82.4)	36 (72)	358 (83.7)	89 (75.4)	305 (84.7)
Strongyles	392 (82)	36 (72)	356 (83.2)	89 (75.4)	303 (84.2)
Strongyles + *Strongyloides papillosus*	20 (4.2)	4 (8)	16 (3.7)	4 (3.4)	16 (4.4)
Strongyles + *Moniezia* spp.	23 (4.8)	2 (4)	21 (4.9)	10 (8.5)	13 (3.6)
Infection with the three	3 (0.6)	0 (0)	3 (0.7)	0 (0)	3 (0.8)
*Moniezia* spp.	2 (0.4)	0 (0)	2 (0.5)	0 (0)	2 (0.6)
Goats shedding ≥500 strongyle epg	251 (52.5)	21 (42)	230 (53.7)	58 (49.2)	193 (53.6)
Egg count/gram of positive faeces					
Strongyles	1842 (20–31,040)	2325 (40–31,040)	1793 (20–22,080)	1786 (40–31,040)	1858 (20–22,080)
*Strongyloides papillosus*	332 (10–1600)	53 (10–80)	391 (40–1600)	53 (10–80)	391 (40–1600)

In order to identify risk factors with significant effects on the odds of animals included in the study to be positive for strongyle nematodes or on the eggs per gram (epg), multi-variate logistic and negative binomial regression models were fitted, respectively. As potential risk factors, the variables area, season, age group, sex and an interaction between sex and age group were initially considered and step-wise eliminated to optimise the Akaike information criterion (AIC). The final negative binomial regression model showed that the epg of strongyle nematodes was significantly lower in winter than in autumn (rate ratio 0.15; *P* < 0.0001) (Table 3). Moreover, the epgs were significantly higher in both Nyala regions than in Beleil. However, the final model showed only a moderate improvement compared to the Null model since the Nagelkerke pseudo R^2^ value was below 0.2. Logistic regression analysis revealed that again winter season was associated with reduced odds for animals to be infected with strongyles (odds ratio 0.05) (Table 4). The odds to be positive were also increased for Nyala Domaia compared to Beleil while other regions did not differ significantly although *P* values were only slightly above 0.05 but 95% confidence intervals (CIs) did not include an odds ratio of 1. Surprisingly, young animals (i.e. < 1 year) significantly less often shed strongyle eggs than adults (Table 4). Again, there was only a moderate improvement of the model in comparison to the Null model.

**Table 3:** Final negative binomial regression model to identify variables with influence on egg counts

	Estimate	Standard error	*P* value	Rate ratio	95% confidence interval
Intercept	7.218	0.245	< 0.0001		
Season: autumn vs.					
Winter	−1.910	0.244	< 0.0001	0.148	0.092–0.240
Study area: Beleil vs.					
Nyala, Domaia	0.657	0.297	0.023	1.929	1.052–3.395
Nyala, Majok	1.147	0.479	0.017	3.150	1.252–8.311
Kass	0.181	0.281	0.521	1.198	0.670–2.033

Nagelkerke R^2^ = 0.114

**Table 4:** Final logistic regression model to identify variables with significant effect on the odds of animals to shed gastrointestinal nematode eggs

	Estimate	Standard error	*P* value	Odds ratio	95% confidence interval
Intercept	2.451	0.458	< 0.0001		
Season: autumn vs.					
Winter	−2.980	0.632	< 0.0001	0.051	0.012–0.152
Study area: Beleil vs.					
Nyala, Domaia	1.502	0.731	0.034	4.489	1.128–22.072
Nyala, Majok	1.674	0.869	0.054	5.333	1.022–33.109
Kass	−0.886	0.475	0.062	0.413	0.148–0.983
Age: adult vs.					
Young	−0.867	0.287	0.002	0.420	0.239–0.739

Nagelkerke R^2^ = 0.164

### Albendazole efficacy in goats naturally infected with gastrointestinal nematodes

Table 5 summarises the efficacy of ABZ in each study area in terms of FECR and EC_50_ values with 95% CIs. Albendazole efficacy was evaluated using two different oral doses (5 or 10 mg ABZ/kg bw), at two different days (eight and 14 post treatment), with two different calculation methods (paired and unpaired) using Baysian models with zero-inflation implemented in the eggCounts package version 1.1–1 for R on the online platform (http://shiny.math.uzh.ch/user/furrer/shinyas/shiny-eggCounts/) [17]. Using the 5 mg ABZ/kg bw dosage, data from Beleil revealed full susceptibility to ABZ no matter if an unpaired comparison with a control group or a paired comparison with the same animals before treatment was used. It was also irrelevant if post treatment samples were collected on day 8 or 14. This was in agreement with an EC_50_ value in the egg hatch test of 0.06. In contrast, the 5 mg ABZ/kg bw dosage was not effective in Nyala Domain and Kass with all analysis methods used and the EC_50_ value in the egg hatch test was also above 0.1 μg thiabendazole/ml. Retreatment of goats that still had an epg ≥500 on day 14 post treatment with a second dosage of 5 mg ABZ/kg only slightly decreased epgs and calculated FECRs ranged between 18 and 62% (Table 5). Treatment of goats with the 10 mg/kg bw dosage indicated ABZ resistance in Nyala Domain using post treatment day 14 data irrespective of the analysis method while day 8 data suggested resistance only when unpaired analysis was used whereas paired analysis was inconclusive. Similarly, inconclusive FECR values were obtained for a 10 mg ABZ/kg bw dosage in Kass for day 8 post treatment (both in paired and unpaired analyses) while day 14 data indicated resistance (Table 5). Remarkably, the EC_50_ value in the egg hatch test for this parasite population was 0.12 μg thiabendazole/ml, which is also only slightly above the threshold for resistance of 0.1 μg thiabendazole/ml. In Nayla Majok, only one treatment group was available and data obtained on day 8 and day 14 post treatment were contradictory suggesting susceptibility at the earlier time point and resistance at the later. However, the relatively high EC_50_ value of 0.18 μg thiabendazole/ml also suggested that resistance is present in Nyala Majok (Table 5).

**Table 5:** Faecal egg count reduction (95% confidence intervals), and EC_50_ (95% confidence intervals) in the egg hatch test, with goats naturally infected with gastrointestinal nematodes at Nyala (Domaia and Majok), Beleil and Kass, South Darfur State, Sudan, and treated with 5 or 10 mg/kg body weight albendazole

Study area	Season	GI nematodes	Dose and No. of animals in each trial	Day 8		Day 14		EC_50_ (μg thiabendazole /ml)
				FECR (%) unpaired^a^	FECR (%) paired^a^	FECR (%) unpaired^a^	FECR (%) paired^a^	
Nyala, Domaia	Autumn	Strongyles	5 mg/kg Control: n = 30, treated: *n* = 53	75 (61–83)	72 (71–73)	72 (61–79)	69 (68–70)	
			5 mg/kg Control: n = 10, retreated^b^: n = 25	62 (22–80)	34 (29–37)	48 (11–72)	18 (13–22)	0.24 (0.04–1.52)
			10 mg/kg Control: n = 10, treated: *n* = 17	90 (84–94)	92 (91–93)	80 (53–90)	84 (83–85)	
	Winter	Strongyles	10 mg/kg Control: *n* = 6, treated: *n* = 8	85 (64–93)	87 (84–89)	75 (48–89)	83 (80–86)	0.18 (0.14–0.24)
Nyala, Majok	Winter	Strongyles	10 mg/kg Treated: *n* = 10	n.a.^c^	94 (92–95)	n.a.^c^	86 (84–88)	0.18 (0.04–0.81)
Beleil	Autumn	Strongyles	5 mg/kg Control: n = 10, treated: *n* = 30	95 (92–97)	96 (95–96)	95 (93–97)	95 (94–95)	0.06 (0.02–0.17)
		*Strongyloides papillosus*	5 mg/kg Treated: n = 3	n.a.^c^	100	n.a.^c^	100	
Kass	Autumn	Strongyles	5 mg/kg Control: n = 15, treated: *n* = 21	89 (82–94)	92 (91–92)	77 (63–85)	74 (73–75)	0.12 (0.02–0.69)
		*Strongyloides papillosus*	5 mg/kg Control: *n* = 4, treated: n = 4	100	100	100	100	
		Strongyles	10 mg/kg Control: n = 15, treated: *n* = 15	91 (83–95)	94 (94–95)	87 (77–91)	87 (86–88)	

^a^FECRs were calculated either by comparing data post treatment between treatment and control group (unpaired) or between data post and pre treatment (paired)

^b^Retreated goats were initially treated first with 5 mg/kg albendazole and received a repeated dose of albendazole (5 mg/kg body weight) on day 14

^c^No control group available since less number of positive animals were detected

Although data for *S. papillosus* should be considered with caution due to the small number of positive animals treated (*n* = 7), the 100% efficacy that was observed suggests that there is no problem of ABZ resistance in this parasite species (Table 5).

Coprocultures followed by genus differentiation of L3 revealed that the predominant parasite before treatment was *Haemonchus* spp. in addition to moderate or low frequency of *Trichostrongylus* spp. and *Oesophagostomum* spp./*Chabertia* spp. (Table 6). In contrast, when faecal samples collected on day 10 post treatment were used for coprocultures, only *Haemonchus* spp. L3 were detected.

**Table 6:** Pooled faecal cultures for differentiation of strongyle third stage larvae in goats naturally infected with gastrointestinal nematodes at Nyala (Domaia and Majok), Beleil and Kass, South Darfur State, Sudan, before and after oral administration of 5 or 10 mg/kg body weight albendazole to the treated groups

Strongyle nematodes	Nyala								Beleil^a, c^		Kass^a, b, c^	
	Domaia^a, c^		Domaia^b, c^		Domaia^b, d^		Majok^b, d^					
	Day 0	Day 10	Day 0	Day 10	Day 0	Day 10	Day 0	Day 10	Day 0	Day 10	Day 0	Day 10
*Haemonchus* spp. (%)	75	100	83	100	99	100	90	100	96	100	91	100
*Trichostrongylus* spp. (%)	24	0	14	0	1	0	10	0	2	0	4	0
*Oesophagostomum* spp.*/Chabertia* spp. (%)	1	0	3	0	0	0	0	0	2	0	5	0

^a^Goats treated with 5 mg/kg body weight albendazole

^b^Goats treated with 10 mg/kg body weight albendazole

^c^Trial conducted in autumn

^d^Trial conducted in winter

### Albendazole efficacy in goats experimentally infected with *Haemonchus controtus*

Since results of faecal egg count reduction test (FECRT) in the field reveald that ABZ at 5 or 10 mg/kg bw was not fully effective against *Haemonchus* spp. populations from Nyala and Kass, this finding was confirmed using experimentally mono-infected animals. For this purpose, infective L3 were obtained by collecting adult female *H. contortus* from slaughtered goats, homogenisation to release eggs and coproculture. In general, FECR was lower than in the field tests (range 35–78%) and resistance was evident indepedently of the day used for resampling post treatment or if the treatment group was compared with a control group (unpaired) or with its own epgs before treatment (paired) (Table 7). The calculated EC_50_ values for both populations in the egg hatch test was 0.13 and 0.15 μg thiabendazole/ml to Nyala and Kass (Table 7) and thus higher than the threshold for resistance of 0.1 μg thiabendazole/ml.

**Table 7:** Faecal egg count reduction (and 95% confidence intervals) and EC_50_ (and 95% confidence intervals) in the egg hatch test with male goats experimentally infected with *Haemonchus contortus* isolates collected from local abattoirs of Nyala and Kass, South Darfur State, Sudan, before and after oral administration of 5 or 10 mg/kg body weight albendazole to the treated groups

Study area	Dose	Day 8		Day 14		EC_50_ (μg/ml thiabendazole)
		FECR (%) unpaired^c^	FECR (%) paired^c^	FECR (%) unpaired^c^	FECR (%) paired^c^	
Nyala	5 mg/kg^a^	78 (54–88)	77 (76–81)	62 (11–81)	35 (27–42)	0.13 (0.05–0.34)
Kass	5 mg/kg^a^	76 (18–91)	77 (73–79)	70 (28–88)	56 (52–61)	0.15 (0.07–0.33)
	10 mg/kg^b^	n.a.^d^	77 (75–80)	n.a.^d^	82 (80–85)	

^a^*n* = 16 (8 for each group, treated and control)

^b^*n* = 8

^c^FECRs were calculated either by comparing data post treatment between treatment and control group (unpaired) or between data post and pre treatment (paired)

^d^No control group available since the initial control group was treated with 10 mg/kg albendazole

The mean (and 95% CI) epg faeces of both natural and experimental infection trials before (day 0) and after ABZ administration (day eight and 14) are presented in Additional file 2: Table S1.

## Discussion

The design of the present study focused on the detection of potentially present anthelmintic resistance in gastro-intestinal helminth of goats. The FECRT results using naturally and experimentally infected animals at a 5 mg/kg bw or a double 10 mg/kg bw oral dose of ABZ in two different seasons as well as the egg hatch test data in addition to coprocultural examinations showed for the first time that benzimidazole resistant *H. contortus* were present in goats in South Darfur. This is also the first published report on the occurrence of anthelmintic resistance in Sudan. Although one might expect that under subsistence farming conditions, use of anthelmintics is less frequent than on commercial farms with high stock numbers and high density of animals, the finding of resistance in Sudan extends the reported presence of resistant *H. contortus* in many different poor tropical countries including several in Africa [18, 19] such as Ethiopia, Kenya, Mozambique, Tanzania and Uganda [20–24]. Indeed, recent data from questionnaires suggests only moderate treatment frequencies in the study area [11], which are probably not sufficient to explain widespread ABZ resistance, in particular since ivermectin was also used.

At a 5 mg/kg bw or a double 10 mg/kg bw oral ABZ dose and in both field and experimental infection trials, GIN populations from two study areas, Nyala and Kass, showed insufficient efficacy of ABZ. Both unpaired and paired FECRT analyses using epg data obtained on day 14 consistently identified these populations as resistant according to the World Association for the Advancement of Veterinary Parasitology (WAAVP) criteria that use a FECR cut-off value of 95% for resistance detection [25, 26]. In comparison, the FECRT was more frequently inconclusive on day 8 than on day 14 post treatment. Remarkably, there is a recent publication on the optimal time-point of post benzimidazole treatment sampling for human *Ascaris lumbricoides* infections that also suggests that a later time point might be preferable [27] albeit in this case the FECR was underestimated on day 8 and not overestimated as in the present study.

The ABZ dosage of 5 mg/kg bw is recommended for sheep but is widely used for goats as well in Sudan (Mohammedsalih, personal observation). However, goats should be treated with a double dosage since they metabolise most anthelmintics much faster than sheep [14]. Therefore, it must be assumed that under-dosing is a widespread problem in the study areas and it is well known that this can lead to rapid selection of resistance [18, 28]. If the FECRT was performed using the double dosage of 10 mg/kg bw, the observed FECRs slightly increased by 0–26% depending on the trial type, study day and the chosen control data. However, they were still below the criteria defined by the WAAVP as threshold for resistance, in particular on day 14 post treatment. These data exclude that the resistant phenotype was only detected in the FECRT due to the low dosage of 5 mg/kg bw used. The local habit to deworm the goats twice with the sheep dosage within 2 weeks does apparently not improve the anthelmintic efficacy as revealed by the data where two consecutive treatments were conducted in Domaia. This second treatment resulted in a FECR of only 18% (paired test on day 14 post treatment) showing that it barely affected the resistant parasite populations while presumably selecting efficiently against susceptible worms. The application of the same amount of anthelmintic (and the same or even a smaller amount of money due to lower logistical efforts) by giving a single treatment using twice the sheep dose would appear highly advisable. In other studies conducted in areas where goats are frequently treated with the ovine benzimidazole dose (i.e. 5 mg ABZ/kg bw), doubling the dose also improved the efficacy by 13–18.4% in herds infected with benzimidazole resistant GINs [29, 30]. The egg hatch test results further supported the results of the FECRT. The EC_50_ ranged from 0.12 to 0.24 μg thiabendazole/ml. This range was also higher than the value set as threshold by the WAAVP (0.1 μg thiabendazole/ml) [25].

As in South Darfur, *H. contortus* is the main nematode species involved in anthelmintic resistance of small ruminants in several other countries [29, 31, 32]. The much higher reproductive potential of *H. contortus* possibly explains why resistance has developed first in this species rather than in *Trichostrongylus* spp. and *Oesophagostomum* spp.*/Chabertia* spp. [33]. It is interesting that in one area, Beliel, a 5 mg/kg bw dose of ABZ was still effective against *H. contortus* (95% FECR) and that also the EC_50_ in the egg hatch test was below the threshold for resistance.

The reason that *H. contortus* developed resistance in South Darfur is possibly related to the frequent under-dosing while high treatment frequencies and/or a high number of anthelmintic treatments using the same anthelmintic drug class for years at least appear not to be a widespread problem [11]. However, that does not exclude that high treatment frequencies with benzimidazoles can be involved locally on certain farms. In this state, ABZ has been used for more than two decades with an average of three treatments per year, particularly also including treatments in the dry seasons [10, 11]. Other reports also described anthelmintic resistance development in herds treated with ABZ two to 10 times per year [34, 35]. With 8 months of no rain, strongyle larvae are unlikely to survive in the ground, which eliminates any refugium on pasture. Therefore, treating animals during the dry season will further decrease the refugium with the effect that the next generation of worms is only the progeny of worms surviving treatment. This effect can already be seen in the data of Nyala Domaia where some animals were treated twice with 5 mg/kg bw ABZ dose and the FECR did not decrease considerably after the second treatment. Another factor potentially contributing to the selection of resistant worm populations is that not all anthelmintics used in Sudan may be genuine. This may mean that they actually contain considerably less drug than they claim to contain [36, 37] and this will further worsen under-dosing. This is why all the ABZ for the trials described here was imported from Europe. The practice of anthelmintic treatments without proper prior estimation of animal body weight has also resulted in under-dosing, which accelerates the frequency of resistance development [38]. Indeed, a typical local treatment regimen would involve treatment of all young animals with e.g. 125 mg/animal and all adults with 250 mg/animal without any aiming to determine or estimate the body weight (Mohammedsalih, personal observation). Even if the dosages would be optimal for the mean body weight of the groups, this might result in under-dosing in the heavier animals. This under-dosing in a considerable fraction of the host population would lead to selection of resistant parasites, which is not really influenced by over-dosing in the animals with the lowest weight.

The egg counts of goats showed a wide variation (see epg ranges in Table 2) indicating that not all animals need treatment. Therefore, so-called targeted selective treatment (TST) approaches would be advisable since they reduce the amount (and costs) of drugs administered and decrease selection of resistant genotypes. In South Africa, ranking of the colour of the mucous membrane of the eye using the so-called FAMACHA chart has been successfully evaluated as an indicator of *H. contortus* burden in sheep. The FAMACHA approach can be used even by illiterate individuals to specifically identify sheep requiring treatment instead of whole herd treatment and thus reducing anthelmintic use [39]. Since the burden of GIN in South Darfur for goats is predominantly caused by *H. contortus*, this method could well be applied also in the study area. It has been shown that TST can slow the development of resistance [39, 40]. The next steps regarding worm control in South Darfur State should involve training pastoralists to use FAMACHA to decide which animals require treatment and identifying where resistance is present so that a change of anthelmintic from ABZ to other classes of anthelmintics can be recommended. Furthermore, the genetic background of the resistance should be clarified based on the quantitative analysis of the β-tubulin isotype 1 gene of *H. contortus* [41]. This approach has been shown to be suitable to detect benzimidazole resistance in large field studies [42, 43] but this has so far in Africa only been performed once for *H. placei* in cattle from Nigeria [44].

In addition to the investigation of the occurrence of anthelmintic resistance, this study also investigated aspects concerning the epidemiological situation of gastrointestinal helminth infection in South Darfur. However, the obtained data about infection prevalence and intensity in the present study are not representative since convenience sampling was applied, the areas were visited at most in two seasons and parameters such as type of pasture, animal weight and health status or local humidity and temperatures were not recorded. Thus, risk factors that were identified in the present study are therefore not representative for the whole goat population in South Darfur. Nevertheless, the analysis of risk factors within the study animals provides at least some preliminary insights into geographical and seasonal information of the gastrointestinal helminths that infect goats in South Darfur. Additionally, the correlation of the infection rate with animal age and sex were investigated. In autumn, the frequency of infection was high (86.6%), which is due to the favourable environmental conditions, in particular high humidity that favours survival of infective larvae on pasture [45]. The development and growth of most helminth species requires warm and wet conditions that make hatching of eggs possible and improves the development of eggs to L3 [46]. With low humidity in winter (28.4%) in South Darfur, infection from pasture may not be possible. Therefore, goats that tested positive in this season may still be harbouring helminths from the autumn. This assumption is in agreement with previous reports from South Darfur [5, 47]. Three helminth egg types were differentiated in the faeces of the tested goats: Strongyles, *S. papillosus* and *Moniezia* spp. The prevalence of strongyles was by far the highest (82%) and when the harvested L3 from pooled faecal cultures were identified microscopically, three genera/groups were found, *Haemonchus* spp., *Trichostrongylus* spp. and *Oesophagostomum* spp.*/Chabertia* spp., with *Haemonchus* spp. representing the by far most abundant larvae. However, it is impossible to conclude the number of adult parasites in the goats from these data since different worm species show strong differences in fecundity and *Haemonchus* spp. are among the species producing the highest amounts of eggs. Another confounder is the development rate of larvae in culture, which might differ between parasite species. Similar compositions of larval populations in cultures have been reported previously from goats in South Darfur [5] and from other tropical regions [48, 49]. Absence of other GIN in the tested goats (e.g. *Cooperia* spp., *Gaigeria* spp., and *Trichuris* spp.) is in agreement with the low infection rate with these helminths in the study area reported previously [5]. The study has indicated significant differences in the prevalence of strongyle egg shedding between adult and young goats, surprisingly with a lower value in the latter (84.7% vs. 75.4%). In two different studies from Ethiopia [46] and Sudan [5], no significant effects of age on prevalence of strongyles in goats was observed. However, other studies indicated the opposite effect as observed here, i.e. young goats showed a higher prevalence of strongyle eggs in faeces when compared to adults [48, 49]. These differences may be attributed to different husbandry systems that are practiced in each study area. In Sudan, the delivery of goats is more frequent in autumn when compared to winter and summer [50]. This means most of the born kids would become infected with GIN in the next autumn when they are young adults. Another factor that may affect the frequency of infection with GIN in young goats in South Darfur is the fact that some farms graze kids in separate groups than adults, to keep milk for human consumption. Neither sex nor a potential interaction between sex and age group included to detect potential effects of sex only in adult, sexually active, goats had a significant influence on the odds to be positive or on the epg in the regression analysis. However, it must be stated that the number of male goats in the study population was very low and the study therefore probably did not have the power to detect such effects if they would be present.

## Conclusions

The study provided evidence for the occurrence of benzimidazole resistance in goats in two out of three different South Darfur (Sudan) study areas (Nyala and Kass) in goats naturally infected with GINs. This finding was confirmed by using goats experimentally infected with local *H. contortus* populations from Nyala and Kass. Consistent use of ABZ in goats at 10 mg/kg bw as well as the implementation of TST approaches in South Darfur State are urgently needed to overcome the development of anthelmintic resistance. This investigation furthermore provides epidemiological data on GIN infections demonstrating that GIN infections are highly prevalent in the three tested areas, with *Haemonchus* spp. being the most predominant parasite identified.

## Methods

### Study area

South Darfur State, southwest Sudan, lies between latitude (Lat.) 11°30′N and longitude (Long.) 24°40′E and has a size of about 127,300 km^2^ (Additional file 1: Figure S1). The climate is a savannah where open grazing occurs with a clay-sandy soil in the south and a sandy soil in the north. The pasture is mainly dominated by abo-asabei grass (*Dactyloctenium aegyptium*), but also several legumes are present. South Darfur is characterised by the presence of very long draught season with no rain at all and only a single rainy season in autumn (July – October). According to the meteorological data obtained from Nyala Airport Meteorological Station [51] between 2013 and 2015, mean rainfall throughout autumn was 459 mm (range 377–546 mm). In autumn, mean minimum and maximum temperatures of 24.7 and 37.6 °C, and 54.8% mean relative humidity were observed. In winter (November – February), the weather is dry and temperatures are lower with mean minimum and maximum temperatures of 17.3 and 34.5 °C, and mean relative humidity of 28.4%. In summer (March – June), the weather is dry and hot, the mean minimum and maximum temperatures are 24.1 and 41 °C, with mean relative humidity of 20.5%.

Three South Darfur study areas were investigated for the presence of gastrointestinal helminths: Nyala, Beleil and Kass (Additional file 1: Figure S1). Nyala city (Lat. 12°05′N; Long. 24°88′E) is the Capital of South Darfur, Beleil (Lat. 12°02′N; Long. 24,99′E) is a town 12 km east of Nyala and Kass (Lat. 12°50′N; Long. 24°28′E) is 82 km northwest of Nyala. In Nyala, two villages were selected: Domaia to the west and Majok to the east of Nyala. The criteria for selection of these areas were the high density of goats and the popularity of their livestock markets.

### Study animals

Since farmers in South Darfur sell or slaughter goats at any time, a minimum flock size of five was chosen to avoid that no treated goats are present on the farm when revisiting the animals eight and 14 days post treatment. A second inclusion criterion was the absence of anthelmintic treatment for 30 days. Faecal samples from 478 male and female goats of different ages were collected and tested for the presence of gastrointestinal helminths in two different seasons, autumn (June – November, 2015 and 2016) and winter (December 2015 – January 2016). Goats (*Capra aegagrus hircus*): In South Darfur the predominant rearing breed is a desert goat, but cross breeds are also widespread. Since many farmers in Nyala, Beleil and Kass rear animals inside their houses, the number of goats per farm is low (2–30 heads including kids) but a few farms have larger numbers of animals. In these areas, goats are grazing during the daylight on pasture which are shared with other species, in particular cattle and sheep, while at night animals are gathered at small pens inside the house*.* Individual goats were selected for screening by convenience.

### Drug treatment

Albendazole, commercial brands (Endospec 2.5%, Bimeda, Batch No: XMF031A; Valbazen 1.9%, Elanco, Batch No: 5138888; and Albex® 10% w/v oral suspension, Chanelle, Batch No: H30275), was imported from Europe. To make our recommendations highly acceptable to the veterinary authorities and farmers in Sudan, ABZ was given orally at 5 mg/kg bw, which is the recommended dose for goats in Sudan, twice at the recommended dose after 14 days as commonly practiced in the region, and at the double dose, i.e. 10 mg ABZ/kg bw as recommended usually for goats due to their higher drug metabolism. Goat weights were determined using a spring balance scale (100 kg upper limit) for animals aged up to 12 months. For adults, the linear body measurement approach was used to estimate the weight based on the measure of heart girth and body length (in centimetre). The estimated body weight was calculated using the formula [52]:

$$ \mathrm{Heart}\ \mathrm{girth}\times \mathrm{heart}\ \mathrm{girth}\times \mathrm{body}\ \mathrm{length}/600=\mathrm{animal}\ \mathrm{weight}\ \mathrm{in}\ \mathrm{kilograms} $$

### Albendazole efficacy in goats naturally infected with gastrointestinal nematodes

In the three screened areas (Nyala: Domaia and Majok, Beleil and Kass) goats positive for an infection with GINs and shedding ≥500 strongyle epg faeces were selected. The cut-off value (≥500 epg) was used according to some previous studies which preferred evaluating anthelmintic resistance in sheep and goats with moderate to highly GINs infection rate [53, 54] and the fact that the precision of the FECR estimate increases with the number of counted eggs [55] which can be more easily achieved using animals with high egg counts. Animals were labelled and grouped into control and treated group based on age class (< 12 months old and ≥ 1 years old), followed by epg (500–1000, 1000–2000, 2000–3000, …) within each age class. Then, animals from each group were assigned arbitrarily to each experimental group. This was done aiming to achieve a ratio of control/treatment animal of 1:2 if > 30 animals were available or of 1:1 if the number of goats was lower. In addition, animals were assigned to groups with the aim to have animals from each farm present in all experimental groups. The goats of the treatment groups individually received 5 mg/kg bw (104 goats: Domaia (53), Beleil (30) and Kass (21)) or 10 mg/kg bw ABZ (50 goats: Domaia (25), Majok (10) and Kass (15)). As farmers in South Darfur preferred to retreat sheep and goats with anthelmintics (i.e. ABZ) 2 weeks after first treatment (Mohammedsalih, personal observation), goats (*n* = 25) treated with 5 mg/kg bw ABZ in Domaia that continued shedding ≥500 strongyle epg were retreated on day 14 with the same dose. Animals in each group were tested first before treatment (day 0) and then eight and 14 days after treatment. All goats involved in the study of ABZ efficacy in naturally infected animals stayed in their flocks throughout the experiment and remained there until after the experiment was finished.

### Albendazole efficacy in goats experimentally infected with *Haemonchus contortus*

After ABZ efficacy was evaluated in trials with naturally infected goats, only *H. contortus* larvae were identified in pooled faecal cultures of animals post treatment. Therefore, the following experimental trials were designed and conducted at the premises of the Faculty of Veterinary Science, University of Nyala. Two separate trials were conducted using two different *H. contortus* isolates from two different South Darfur study areas, Nyala and Kass. For each trial, abomasa from 50 goats naturally infected with *H. contortus* were collected from the respective local abattoirs. Mature gravid female *H. contortus* were isolated, crushed, pooled and cultured in heat-treated bovine faeces. For this purpose, faeces from two GIN-free calves were dried and heated to 70 °C for 2 h. Before culturing, the heat-treated faeces were moisturised using distilled water. Harvested eggs were added and mixed with faeces before incubation at 22–27 °C for 8 days. The infective third stage larvae (L3) from each culture were harvested using the Baermann method and the number of L3 per ml of each culture harvest was determined [56]. Three weeks prior to each infection trial, 16 healthy male goats (3–6 months old) were weighed (6–18 kg) and treated with levamisole (8 mg/kg bw, Ripercol® drench, Elanco, Batch No: 13KQ054). During the trials, goats were fed dry hay with free access to drinking water, and care was taken to avoid any contamination of pens with nematode larvae from outside. Each goat in the two trials received 150 L3/kg bw orally [57], and then half were treated with ABZ at 5 mg/kg bw on day 23 post infection and half were left as untreated controls. The no-drug control group of the Kass-isolate received 10 mg/kg bw ABZ on day 14 of the initial phase (i.e. day 37 post infection) after the initial trial with 5 mg/kg bw was finished. Faecal egg counts were determined for each trial before treatment (day 0) and then eight and 14 days after ABZ administration. Goats from the experimental infections were slaughtered after the experiments were completed, respecting withholding times for the drugs. Goats were slaughtered at Nyala abattoir according to the local standard procedures without stunning but using a deep incision of the throat opening both carotid arteries and jugular veins (halal method) [58].

### Coproscopic analysis

Faecal samples were collected directly from the rectum of individual animals in plastic bags, labelled and kept at 4 °C for a maximum of 24 h before counting using the Mini-FLOTAC technique with a detection limit of 5 epg [59]. Samples from Nyala and Beleil were analysed at the laboratory of Parasitology, Faculty of Veterinary Science, University of Nyala, while samples from Kass natural infection trials were analysed directly in the field.

### Egg hatch test

Fresh, pooled faecal samples were collected for the egg hatch test from natural and experimental infection trials on day 0 and directly used within 4 h. The test was performed as recommended by the WAAVP [25, 60]. Concentrations of thiabendazole (Sigma Aldrich) dissolved in dimethyl sulphoxide were used at final concentrations in the well of 0.5% DMSO and 0.00, 0.05, 0.1, 0.2, 0.3 and 0.5 μg/ml thiabendazole. Fresh eggs were inspected microscopically to confirm that embryonation had not begun yet when tests were set up. Then, a suspension of approximately 150 strongyle eggs/2 ml was added to each well of the 24-well plates. The plates were incubated at 27 °C for 48 h and the reaction was terminated by adding 10 μl of Lugol’s iodine to each well. The number of eggs and hatched larvae was counted in each well and mean percentages of hatching were calculated from two replicates.

The data was analysed by a four parameter logistic regression model using GraphPad Prism version 5.03 software to determine the concentration of thiabendazole that inhibited 50% of larvae hatching (EC_50_). A worm population was classified as resistant to benzimidazole if the EC_50_ value was higher than 0.1 μg/ml thiabendazole [25, 26].

### Coproculture

Pooled faecal samples were collected from the selected naturally infected goats in Nyala (Domaia and Majok), Beleil and Kass on day 0 and on day 10 post treatment. Larval cultures were prepared and incubated at 22–27 °C with daily moisturising with sterile water for 8 days. The cultures were harvested and the first 100 or (if less than 100) all L3 were identified morphologically (by genera) according to Bowman [56].

### Risk factor analysis

The R software version 3.3.1 and the graphical user interface RStudio version 1.1.383 were used for data analysis. Logistic regressions were calculated using the glm function while negative binomial regressions for egg counts were calculated using glm.nb from the MASS package. As explanatory variables, the season (autumn vs. winter), the sex, the age group (young animals vs. adult) and an interaction between sex and age group were initially considered. Dentition was used to group goats into juveniles (< 12 months old) and adults (≥1 years old) [61]. After calculating a full model with all potential explanatory variables mentioned above, variables were backward eliminated with the aim to improve the AIC using the drop1 function. For the final models, odds and rate ratios with 95% CIs were calculated by applying the confint function on the model coefficients. The Nagelkerke pseudo R^2^ values were calculated using RsqGLM function from the modEvA package.

### Calculation of faecal egg count reduction with confidence intervals

The epg of the animals was used to calculate the efficacy of ABZ based on the FECRT. The FECR was calculated by comparing the treated group epg with the control group epg on days 8 and 14 (unpaired) and by comparing the treated group epg before (day 0) and after treatment on days 8 and 14, respectively (paired) [25, 62]. For this purpose the improved eggCounts package version 1.1–1 by Wang et al. [17] was used. In this version, zero- inflated Bayesian hierarchical models are included, in addition to estimation of the random error of the samples and aggregations between individual hosts in the treatment groups to provide an estimate of the FECR from the mode of the posterior distribution and 95% CI, which was taken as the 2.5 and 97.5 percentiles of the posterior distribution. In this study, zero-inflation was used to improve estimation of FECR with sufficient statistical power.

The efficacy results of the FECRT and anthelmintic resistance status were interpreted as recommended by Lyndal-Murphy et al. [63] and based on the WAAVP methods [25, 64], considering the FECR percentage and upper and lower 95% CI. The efficacy of each treatment was classified as effective, or ineffective or inconclusive. When the percentage reduction of epg and upper 95% CI was equal or more than 95% and the lower 95% CI was equal or more than 90% the drug was considered effective. Drug resistance was present when the percentage reduction of epg and the upper 95% CI was less than 95% and the lower 95% CI was less than 90%. The FECRT result was considered inconclusive when neither of the two other criteria were met.

## Additional files

Additional file 1: **Figure S1.** Study location in South Darfur. 1, Nyala; 2, Beleil; 3, Kass. The map was drawn using Tableau 2018.2.0. (PDF 138 kb)

Additional file 2: **Table S1.** Arithmetic means (and 95% confidence intervals) of eggs per gram faeces. Goats were naturally infected with gastrointestinal nematodes or experimentally infected with *Haemonchus contortus*, in three different South Darfur study areas, Sudan, before and after oral administration of 5 or 10 mg/kg body weight albendazole to the treated groups. (PDF 137 kb)

## Data Availability

All relevant information has been included in the manuscript. Data analysed for this manuscript are available from the corresponding author on request.

## Abbreviations

AIC: Akaike information criterion
bw: Body weight
CI: Confidence intervals
EC_50_: Concentration of thiabendazole that inhibited 50% of larvae hatching
epg: Eggs per gram
FECRT: Faecal egg count reduction test
GINs: Gastrointestinal nematodes
L3: Third stage larvae
Lat: Latitude
Long: Longitude
TST: Targeted selective treatment
WAAVP: World Association for the Advancement of Veterinary Parasitology
