# Supplementary figures and images for: Epidemiology of strongyle nematode infections and first report of benzimidazole resistance in Haemonchus contortus in goats in South Darfur State, Sudan
Source: BMC Vet Res. 2019 Jun 4;15:184. doi: 10.1186/s12917-019-1937-2 (PMC6549335; doi:10.1186/s12917-019-1937-2)

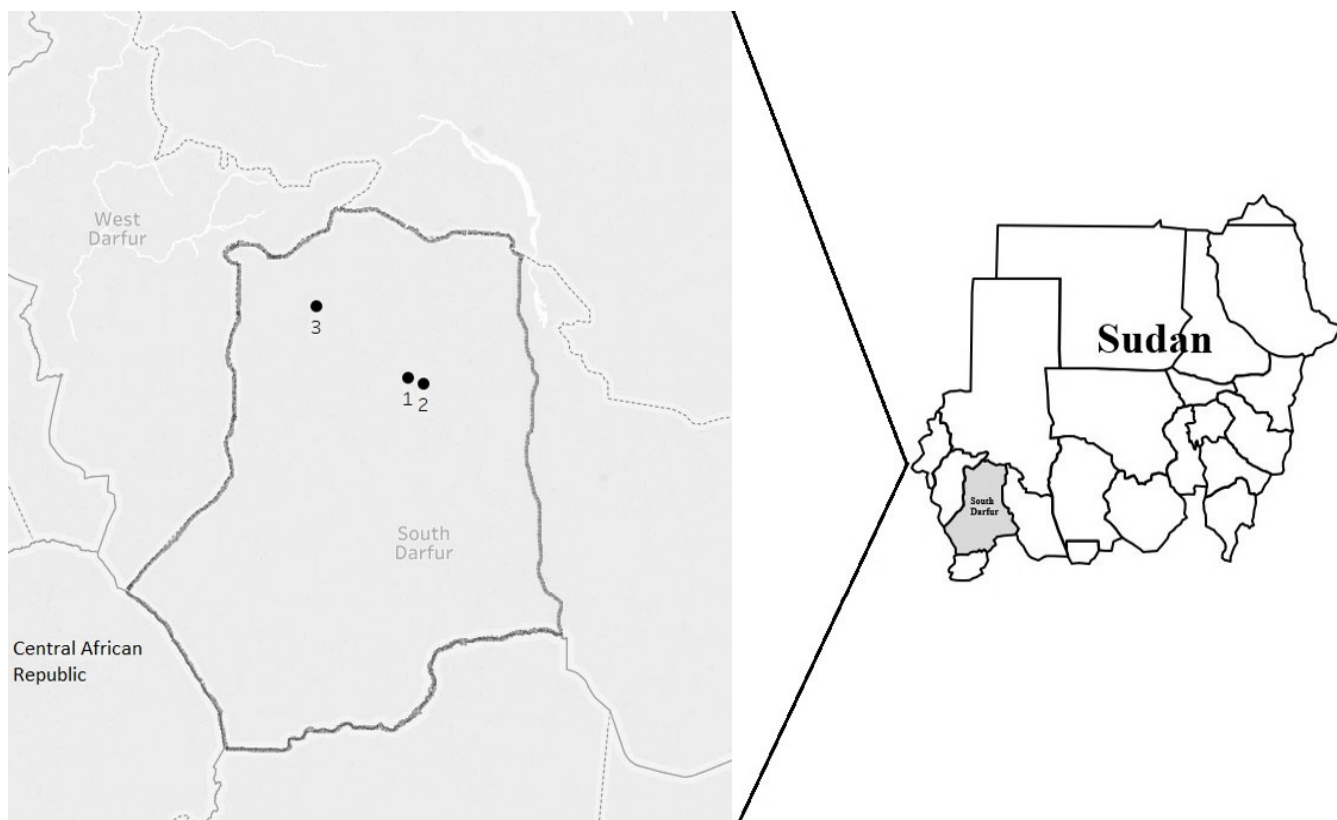

**Fig. S1.** Study location in South Darfur. 1, Nyala; 2, Beileil; 3, Kass. The map was drawn using Tableau 2018.2.0.

Supplement: Supplementary file 1 — Figure S1. Study location in South Darfur. 1, Nyala; 2, Beleil; 3, Kass. The map was drawn using Tableau 2018.2.0. (PDF 138 kb) [file 12917_2019_1937_MOESM1_ESM.pdf]
