# Supplementary material for: Epidemiology of strongyle nematode infections and first report of benzimidazole resistance in Haemonchus contortus in goats in South Darfur State, Sudan
Source: BMC Vet Res. 2019 Jun 4;15:184. doi: 10.1186/s12917-019-1937-2 (PMC6549335; doi:10.1186/s12917-019-1937-2)
Supplement: Supplementary file 2 — Table S1. Arithmetic means (and 95% confidence intervals) of eggs per gram faeces. Goats were naturally infected with gastrointestinal nematodes or experimentally infected with Haemonchus contortus, in three different South Darfur study areas, Sudan, before and after oral administration of 5 or 10 mg/kg body weight albendazole to the treated groups. (PDF 137 kb) [file 12917_2019_1937_MOESM2_ESM.pdf]

Table S1

Arithmetic means (and 95% confidence intervals) of eggs per gram faeces. Goats were naturally infected with gastrointestinal nematodes or experimentally infected with *Haemonchus contortus*, in three different South Darfur study areas, Sudan, before and after oral administration of 5 or 10 mg/kg body weight albendazole to the treated groups.

Study area	Type of infection	Season	Dose	GI nematodes	Groups	Day 0	Day 8	Day 14
						Mean (95% CI)	Mean (95% CI)	Mean (95% CI)
Nyala, Domaia	Natural	Autumn		Strongyles	Control, n=30	3136 (2525 – 3747)	3586 (2826 – 4345)	3511 (2607 – 4414)
					Treated, n=53	3090 (2526 – 3654)	876*# (635 – 1117)	970*# (780 – 1160)
			5 mg/kg	Strongyles	Control, n=10	1072 (739 – 1405)	1800 (1255 – 2345)	1749 (1139 – 2359)
					Treated[a], n=25	1005 (674 – 1336)	666* (341 – 991)	830* (500 – 1160)
			10 mg/kg	Strongyles	Control, n=10	3356 (2216 – 4496)	3642 (2495 – 4789)	3568 (1944 – 5192)
					Treated, n=17	3847 (1088 – 6606)	247*# (133 – 361)	492*# (146 – 838)
		Winter	10 mg/kg	Strongyles	Control, n=6	1000 (614 – 1386)	913 (927 – 1200)	767 (513 – 1021)
					Treated, n=8	1108 (499 – 1716)	143*# (49 – 236)	190*# (64 – 316)
Nyala, Majok	Natural	Winter	10 mg/kg	Strongyles	Treated[b], n=10	1430 (421 – 2439)	76# (12 – 140)	170# (-1 – 340)
Beleil	Natural	Autumn		Strongyles	Control, n=10	2504 (1231 - 3777)	2208 (1163 – 3253)	2404 (1521 – 3287)
			5 mg/kg		Treated, n=30	2215 (1596 – 2833)	73*# (45 – 102)	81*# (53 – 109)
				Strongyloides papillosus	Treated[b], n=3	140 (-184 – 504)	0#	0#
Kass	Natural	Autumn	5 mg/kg	Strongyles	Control, n=15	2880 (1777 – 3983)	3293 (2217 – 4370)	5091 (3515 – 6666)
					Treated, n=21	4034 (2046 – 6023)	335*# (200 – 471)	1046*# (633 – 1458)
				Strongyloides papillosus	Control, n=4	710 (119 – 1301)	910 (-319 – 2139)	1650 (-748 – 4048)
					Treated, n=4	1170 (-1327 – 3667)	0*#	0*#
			10 mg/kg	Strongyles	Control, n=15	2880 (1777 – 3983)	3293 (2217 – 4370)	5091 (3515 – 6666)
					Treated, n=15	4829 (2105 – 7553)	283*# (118 – 447)	633*# (360 – 907)
Nyala	Experimental	Summer	5 mg/kg	*Haemonchus controtus*	Control, n=8	808 (503 – 1113)	1185 (879 – 1491)	1665 (988 – 2342)
					Treated, n=8	940 (153 – 1727)	203*# (38 – 368)	610* (156 – 1064)
Kass	Experimental	Summer	5 mg/kg	*Haemonchus controtus*	Control, n=8	1043 (315 – 1770)	1833 (201 – 3464)	2868 (493 – 5242)
					Treated, n=8	1661 (-140 – 3463)	383*# (-11 – 776)	723* (65 – 1380)
			10 mg/kg		Treated[b], n=8	2178 (257 – 4098)	493# (193 – 792)	385# (83 – 688)

[a]Retreated goats were initially treated first with 5 mg/kg albendazole and received a repeated dose of albendazole (5 mg/kg body weight) on day 14.

[b]No control group available since less numbers of positive animals detected, or the initial control group was treated with 10 mg/kg body weight albendazole.

*Significantly different ($p<0.05$) to control on the same day using a Kruskal-Wallis test with Dunn's post hoc test.

#Significantly different ($p<0.05$) to day 0 in the same group using a Friedman test with Dunn's post hoc test.
